# CMOS Cell Sensors for Point-of-Care Diagnostics

**DOI:** 10.3390/s120810042

**Published:** 2012-07-25

**Authors:** Yekbun Adiguzel, Haluk Kulah

**Affiliations:** 1 METU-MEMS Research and Application Center, Middle East Technical University, Ankara 06800, Turkey; 2 Department of Electrical and Electronics Engineering, Middle East Technical University, Ankara 06800, Turkey; E-Mail: kulah@metu.edu.tr

**Keywords:** point-of-care diagnostics, CMOS, cell sensing

## Abstract

The burden of health-care related services in a global era with continuously increasing population and inefficient dissipation of the resources requires effective solutions. From this perspective, point-of-care diagnostics is a demanded field in clinics. It is also necessary both for prompt diagnosis and for providing health services evenly throughout the population, including the rural districts. The requirements can only be fulfilled by technologies whose productivity has already been proven, such as complementary metal-oxide-semiconductors (CMOS). CMOS-based products can enable clinical tests in a fast, simple, safe, and reliable manner, with improved sensitivities. Portability due to diminished sensor dimensions and compactness of the test set-ups, along with low sample and power consumption, is another vital feature. CMOS-based sensors for cell studies have the potential to become essential counterparts of point-of-care diagnostics technologies. Hence, this review attempts to inform on the sensors fabricated with CMOS technology for point-of-care diagnostic studies, with a focus on CMOS image sensors and capacitance sensors for cell studies.

## 1. Introduction

Expectations lead hospitals to evolve as patient care and disease treatment units rather than diagnosis centers. The aim is to reduce the time required to produce the diagnostic test results (“turn around” time) [1]. This can be achieved by bringing the laboratory tests much closer to the bedside of the patient by erasing the borders of a laboratory [2], which is enabled through the concept of point-of-care diagnostics. The stimulation is the reduced load for patient-care when the transfer of patients is eliminated, which results in paced clinical decision making [3]. This is attained given that, physician's office labs are among the point-of-care settings, other than the hospital emergency rooms, critical-care clinics, satellite labs, (neonatal) intensive-care units, and home testing locations [4]. The importance of diminishing the time scales and revising the patient-doctor interaction profiles can aid the physician in tackling problems associated with the lack of time with patients [5]. Reaching globally district regions with scarce health service access are also amongst indispensable goals to be mentioned here. In resource limited settings, point-of-care cluster of differentiation 4 (CD4) cell-count testing with newly diagnosed human immunodeficiency virus (HIV) infections diminished the loss to follow-up before completing staging, from 57% to 21% [6]. Treatment initiation was thus facilitated. As a result, point-of-care diagnostics and bedside monitoring considerably lessen the need to bring the patient or the specimen to the laboratory, with the ease of access to the diagnostic equipment. These imperatives are pushing the point-of-care diagnostics research forward. The requests for new point-of-care tests from specialized services, to improve the efficiency of their clinical operations [7], is another driving force for supporting the current research in the direction of implementing new techniques in point-of-care diagnosis. Such expectations and wide application draw attention of the market and existing manufacturers. According to a study published in 2010, 26 point-of-care tests with a test volume of 664,287 tests/year is equivalent to 14.5% volume of the central core laboratory of Massachusetts General Hospital (Boston, MA, USA) [7]. In Belgium, implementation of a multi-parameter point-of-care-blood test analyzer was recently reported to reduce central laboratory testing and need for blood transfusions in very low birth weight infants [8]. Besides, it was reported to be cost-efficient for the Belgian national health insurance, with an overall cost reduction of 8.72€ (−8.3%) per neonate, for all birth weight groups [8]. Thanks to similar data, point-of-care immunotesting was presenting a multimillion dollar market at year 2004 and was one of the most rapidly growing sectors in the diagnostics business [9].

For a solid realization of point-of-care diagnostics tools, fast responding, portable, cost-effective, low sample and power consuming, trustworthy, and robust systems that are easy to use and maintain are necessary. The miniaturization capability of the sensors that are produced by micromachining [10] can well be assisting to reach the majority of the listed goals with successful performance for clinical use [11]. Complementary metal oxide semiconductor (CMOS) technology is one of such technologies, which was aided by far the most by the communication and computing technologies [12,13]. Now the clinical services wait for such a contribution. It should be pointed that medical devices may themselves not require the sub-micron scaling of the CMOS technologies, but such developments, which generally serve in the first place for research purposes, can easily be translated into a medical device application. Further, claims of the field are shaped by biological media as the novel resources. Yet, biological media also inherit the trouble of complex nature of the human biology, which is reflected in the diagnostics [14]. Therefore, the integration of CMOS devices with various emerging sensing elements [15], utilizing techniques such as surface activation by chemical means are quiet promising in the field of research involving the development of CMOS-based sensors for point-of-care diagnostics.

For point-of-care diagnostic tests, increasing device size and complexity is mostly reflected in the cost of the biological test, such as in case of fluorescence-activated cell sorting (FACS) analysis by flow cytometry. FACS is the established gold standard for CD4 counting with the use of fluorescently labeled antibody conjugates, with the aid of commercially available equipment [16]. According to the World Health Organization's reports for CD4 T-Cell enumeration technologies, established technologies such as FACSCount (Becton Dickinson, San José, CA, USA) encounter considerable challenges to screen or count thousands of cells due to the requirement for experienced operators and restricted throughput (30–50 samples/day), along with the costs of equipment ($27,000), reagent ($5–20), and maintenance [17]. Motivated by the aim of diminishing device sizes and the related requirements, microtechnologies were applied for blood cell counting as early as 1997 [18], with poor reproducibility due to damage to the electrodes. In 2002, the use of the aperture-impedance method in blood cell counting with a MEMS device having electrodes that were not deteriorated during measurements was reported [10]. These initial efforts were amongst electrical sensing and have been under investigation [19–24] since the appearance of the initial Coulter counter in 1956 [25]. An affinity-chromatography/shear-gradient technique using impedance analysis [26] is under development as a commercial CD4 counting technology [16]. It achieved correlation of the decrease in impedance with cell counts including the threshold for antiretroviral therapy for HIV, which is 350 cells/mm^3^ [16]. However, such advanced technologies also possibly suffer from patient-to-patient variations, multiple wash steps, and low signal-to-noise levels [17]. Methods to produce a single and focused stream of cells without sheath fluid, as a different approach from conventional FACS, have been described mainly through the development of microcytometers [16]. Those include mechanical structures [27,28], dielectrophoresis [29], optical forces [30], hydrodynamic forces [31], electrokinetic transport [32] and ultrasound effect [33]. Conventional technologies can be applied in combination with sensor technologies [34,35], micromachining, and/or microfluidics as well. For instance, bioactivated PDMS microchannels were used to capture human CD4 cell from 0.1 μL of non-diluted whole blood, as a point-of-care method for HIV monitoring [36]. Detection was performed by fluorescence microscopy. Fluorescence imaging is a well-known optical detection method, yet it requires an optical setup and fluorescent tagging of the cells and molecules of interest. Besides, counting under a microscope is required [17] when image processing algorithms are not applied, which requires training of an algorithm, *etc.* Automated imaging cytometry makes use of image processing algorithms and can include CMOS-based sensors [37] or ring resonators [38], associated with immunomagnetic separation [39] or chemiluminescence-based detection [40]. They can be as powerful as flow cytometry but requirements such as blood dilution and staining need to be automated, and the field performance of these systems is yet to be determined [16]. Instead of fluorescence detection or automated imaging, mass detection as an example of mechanical sensing could be an alternative for calculating the amount of target cell or molecule of interest. However, in case of quartz crystal microbalance [41] or cantilever based detection, film formation of the cell layers brings about the risk of interlayer interaction and non-linear thickness evolution [36], when bulky amounts of cells need to be counted. Systems under development for specific applications and rare cell detection are not essentially suitable for cell counting. If cell viability is the case rather than cell counting, field effect transistors for cell viability testing, which can be realized in an industrial CMOS process, is advantageous over conventional patch-clamp technique due to the possibility of real-time, non-invasive, and long-term monitoring of the state of living cells [42]. In general, the technologies and techniques mentioned here do not necessarily compete with the CMOS-based sensors and rather provide a rationale for the involvement of this technology in the point-of-care diagnostics field. Besides, CMOS-based sensors can take advantage of the current CMOS industry to achieve sensor parallelization to improve throughput and mass production, which can even suit for the fabrication of disposable chips as a result of significantly diminished device costs. Extensive use of CMOS-based sensors in DNA diagnostics [43–48] and commercialization of CMOS-based DNA sequencing systems (Ion Torrent Systems) [49] is already promising for the involvement of CMOS-based cell detection systems in medical applications.

The analyte range of point-of-care testing covers the regular components sourced from the blood, plasma/serum, urine, other bodily fluids, feces, and tissue samples of the patient [9]. Considering that the analytes of interest are supplied by unit structures of the organism, namely the cells, the importance of cell sensing in point-of-care diagnosis resides in the responsibility of cell metabolism in providing the basis of health and disease status of the organism. Circulating rare cell detection [50], cell enumeration [10,11,36,51], detection of changes in cellular shapes and functionalities, along with their proteins and biochemical indicators [52–54], and timely monitoring of cellular responses to chemical agents all serve in early diagnosis and proper treatment of the patient. Here, response of cells to drugs, chemicals and toxic agents is included within the concept of point-of-care diagnostic studies. *In vitro* cultures were shown to retain tissue-specific properties, with the use of neuronal network biosensors [55]. Validation of these findings with *in vivo* whole animal studies implies that cell-based studies can replace animal tests in toxicology studies [55]. Thus it can be envisioned that such toxicology measures with the cultured cells of the patients can have potential applications in theranostics, personalized medicine and testing at the point-of-care. The principle lurks behind considering diagnosis and treatment as combinatory tools of patient care [56]. Diagnosis of a disease aids the monitoring of drug therapy as well [3], which is of course valid for point-of-care diagnosis. Also, it should better be reminded that not all point-of-care analyzers are immediately applicable to all testing settings [9].

CMOS-based sensors for cell studies can potentially become indispensable complements of point-of-care diagnostics technologies in the near future. Conventional techniques for cell studies and cell detection require optical and spectroscopic tools, which are found in molecular biology laboratories with cell culture facilities. Investigating blood smears under microscope, cell culturing, DNA amplification and detection, toxicity tests, and immunological approaches are enabled through the emerging biosensor technologies, if not replaced by an innovative method. An analysis of the relevant biosensor literature containing the terms “CMOS” or “integrated circuit” revealed undoubtedly that cell-based sensor types are the most widely published, hence the most popular in practice [57]. Therefore, eliciting their prospective status in the field of point-of-care diagnostics will provide us a snapshot view of the trends in the developing integrated field of health sciences and CMOS-based sensors.

To mention briefly the CMOS technology, it holds the key to attain low-power consumption and small-sized lab-on-a-chip microsystems with integrated microelectronic sensors, in a broad sense. Such sensors consist of sensing elements combined with all the necessary circuitry for signal amplification, filtering, multiplexing, analog-to-digital conversion, communication, and control [12]. Research on biosensors using CMOS currently makes use of mature fabrication processes, which can define features of only ∼0.1 μm or larger [57], yet the industry is working towards systems as small as 22 nm [58]. Among the design considerations, the advantages are offered as miniaturization, low power consumption, massive parallelism, reduction of input-output pins, high sensitivity, scalability, and high reliability of mass production. First of all, high sensitivity is critical for clinical diagnostics. Then, the other advantages are interlinked in the sense that miniaturization facilitates portability and reduces both energy and sample consumption; while it is the low power consumption that mainly allows portability. Besides, massive parallelism helps to achieve multi-sensor arrays with lessened analysis durations; reduction of input-output pins enables easier multiplexing and preprocessing; and scalability allows higher sample throughput [12]. On the other hand, the disadvantages can be exemplified as complex packaging, biocompatibility, and contamination. Complex packaging is due to the necessity of attaining microfluidic functionality at the same time as bonding wire protection [12]. Biocompatibility issue means post-CMOS fabrication and increased final cost, as a result. This issue is bi-sided and has adverse affects for the chip as well, since contamination is highly probable due to direct contact with the samples [12]. In addition, signal detection and isolation remain active areas of research [59].

In the following sections of this review, the *Cell Detection* section starts with *Capacitive CMOS Sensors for Cell Viability Testing*. It introduces the relevant technology based on capacitive coupling of cells on the sensor surface, due to the capacitive behavior of cells that are exposed to weak, low frequency electric fields [60]. *CMOS Image Sensors for Cell Counting* section of *Cell Detection* informs about photonic detection of cells and cellular responses by means of CMOS sensors. It includes a sub-section with the title *Lensfree Ultra-wide-field Cell Monitoring Array Platform Based on Shadow Imaging (LUCAS)*. Then, *Imaging Sensors with Dual Functionalities for Bioscientific Sensing* and *Imaging Sensors for Testing Drug Effects* come as the last sections of *Cell Detection*. Afterwards, *CMOS-Compatible Silicon Nanowire-Based Sensors for Cell Studies* briefly tells about the improved sensing capability and its application for cell sensing by utilizing silicon nanowires as the conducting channels. In the end, a *Conclusion and Outlook* section tries to broaden the perspective of some specific issues that are mentioned in the text. Overall, the aim of this manuscript is to inform the reader about CMOS cell sensors for point-of-care diagnostics and since research generally comes prior to any application or a commercial device, the additional goal is to motivate the people in the field to consider CMOS cell sensors by focusing on their research impact as well.

## 2. Cell Detection

### 2.1. Capacitive CMOS Sensors for Cell Viability Testing

Capacitive sensing based on capacitive coupling can be utilized for cell studies with the use of CMOS sensors. Capacitive biosensors enable label-free detection, which is a desired aspect in point-of-care diagnosis [61]. Studies employing cell viability, morphology and surface attachment monitoring can have implications in biomedicine and biotechnology, as supplementary fields of research for point-of-care diagnostics. For instance, cell viability assessment is crucial for drug screening and biocompatibility characterization of implants. Further, cell-substrate interaction can be used to work on tumor growth, wound healing, and cell migration. These have implications in neuroprosthetics [62].

Prakash and Abshire contributed a lot to the advancement of capacitive CMOS sensors. They have developed CMOS capacitance sensors for cell viability monitoring, whereby cell-substrate capacitive variations that were detected in the fF range [63]. The principle was based on sensing the changes associated with capacitive coupling between an isolated sensing electrode and the cellular matrix, upon alterations of the cell viability and surface adhesion.

**Figure 1.**
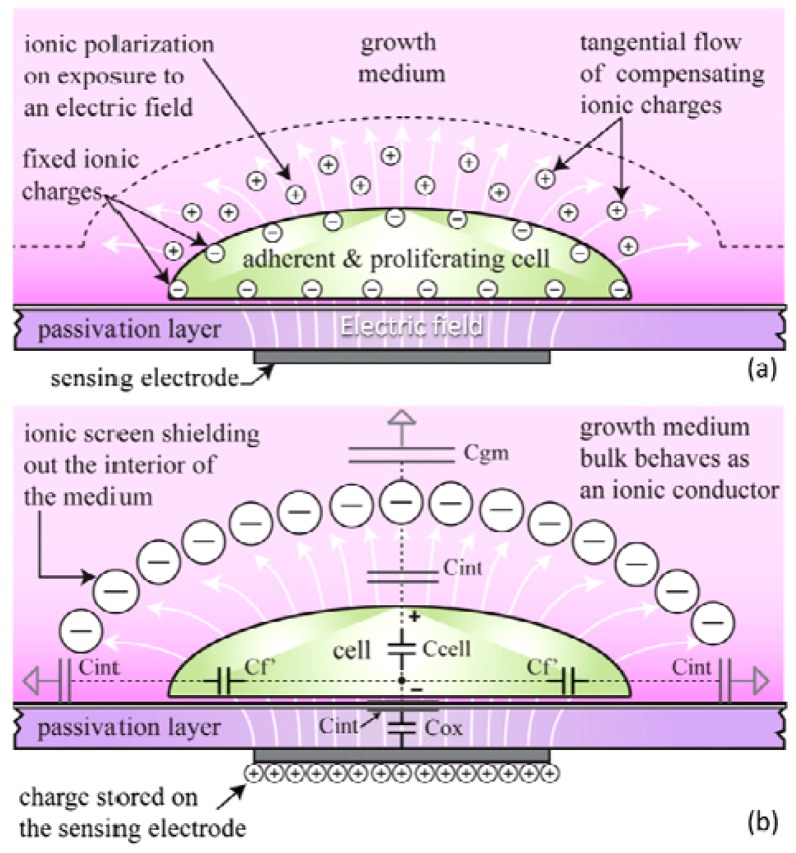
Ionic cloud surrounding the surface-anchored cell over the sensing electrode polarizes (**a**) and leads to the formation of capacitive behavior of the cell, which is contributing to various elements of the sensed capacitance network (b) [60]; (**b**) illustrates the model of sensed capacitance during the adhesion and post-adhesion phase of the interaction process between cell and substrate [64]. C_ox_ stands for passivation layer capacitance, C_cell_ stands for cell layer capacitance, C_f_ stands for fringe parasitic capacitance, C_int_ stands for interfacial capacitance, and C_gm_ stands for growth medium capacitance [60]. Reproduced with the permission of Elsevier.

The capacitance sensor uses the principle of charge sharing and translates sensed capacitance values to output voltages [64]. As a result, the measured capacitance depends on a variety of factors related to the cell, growth medium, and supporting substrate [Figure 1(b)]. Under low frequency electric fields, ionic cloud surrounding the insulating cell polarizes [Figure 1(a)], and the resulting electric dipoles lead to the formation of capacitive behavior of the cell [64]. Capacitance of healthy cells is higher and they adhere stronger to the surface, due to the capacitive coupling between the cells and the underlying electrodes.

Viable cell counting is a tedious task and viability indicators require the use of optic or spectroscopic instruments [65]. CMOS capacitance sensors as viability indicators can provide facile solutions, yet initial studies mainly served as the proof of the concept. Therefore, cell viabilities were first assessed independently and correlated with the capacitance measurement results. Accordingly, Prakash and Abshire [63] prepared their measurement setup data acquisition system inside the incubator, for online monitoring of the sensor responses to cells that were exposed to electric field excitations during the recording intervals. Sensor outputs of bovine aortic smooth muscle cells were followed for 48 h. Viability was assessed in parallel by visual examination of the stained cells in a colorless growth medium, through the characteristic property of healthy cells' taking up and retaining neutral red, contrary to the non-viable cells. The observations were correlated to the sensed capacitance values. By using similar test set-ups, cell proximity to the surface and viability can be studied for cell migration rates within tissue implant scaffolds, which require cells to live, divide, and maybe even transform to specific cell types in a given medium. This approach was used to detect cell proximity to the surface, by utilizing cell-to-surface distance dependence of the capacitance that is formed by the combination of the capacitances between the cell and the passivation layer, and between the passivation layer and the sensing electrode [66]. The relation was inversely proportional with a resolution of 3 nm, and the sensor capacitance resolution was 135 aF.

For on-chip capacitance sensing for cell monitoring, the transducer can be calibrated as a proximity detector by a vertically controlled external metal electrode, with the use of a piezoelectric micropositioner [64]. This was managed with an integrated circuit for sensing the substrate coupling capacitance of anchorage-dependent living cells in a standard culture environment. Capacitance was measured using charge redistribution in response to weak, low frequency electric field excitations.

During adhesion [Figure 1(b)] the interface between the growth medium and the substrate undergoes a drastic change in its structural and dielectric properties resulting in an appreciable variation in the sensed capacitance, with respect to the preadhesion phase [64]. They later applied their cell-substrate capacitance sensing platform for online tracking of human MDA-MB-231 breast cancer cell line proliferation, with a sensor chip that had been fabricated in a 0.5 μm CMOS technology [60]. The sensor registered capacitance increases in the fF range upon cell adhesion and during the forthcoming proliferation [60].

### 2.2. CMOS Image Sensors for Cell Counting

The role of CMOS image sensors since their origin around the 1960s has been changing quiet a lot [67]. Today, CMOS-based image sensors serve as candidate solutions to the problems of counting specific cell types (e.g., CD4 T lymphocyte counts of HIV infected patients [17]) from whole blood. The apparent problems can be grouped in two, first of which is the capture/isolation of cells from whole blood in a high throughput manner and second is the rapid counting of these captured/isolated cells [37]. CMOS based image sensors can participate in actively coping with the latter issue, while the former one is mainly dealing with microfluidics.

Pixel size has a decisive role in case of cell detection by the image sensors that are based on CMOS pixel arrays. In order to improve previous comparable works by scaling down the pixel size, a CMOS image sensor with a sensor of 5 μm pixel size was designed for cell sensing [68]. Further aims were to increase the sensitivity and noise immunity, and to apply simple signal processing without loss of speed and resolution. The authors incorporated a technique that is capable of locating dark objects against a bright background or bright objects on a dark background, for locating multiple cells at a time, without post-capture image processing. This kind of design well suits for integrating with microfluidic actuation in a closed-loop feedback system to form fully implemented lab-on-a-chip systems [68], which can integrate processes [69] and move diagnostics from bench to the bedside [70].

**Figure 2.**
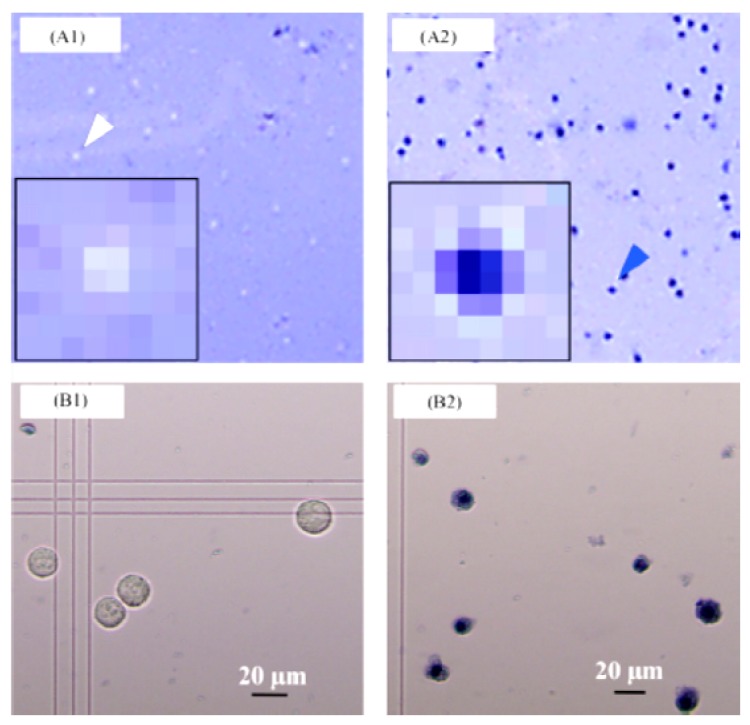
CMOS sensor (**A**) and microscopic (**B**) images of HeLa cells with no (**A1** and **B1**) and with (**A2** and **B2**) heat treatment following trypan blue staining [72]. Reproduced with the permission of Elsevier.

Despite the fact that the resolution of a contact imager is solely determined by its pixel size rather than the size of pixel array [71]; the detection range can be improved to a great extent by staining the cells. Chemiluminescence is also suitable for cell visualization with CMOS image sensors. In order to attain two dimensional (2D) imaging of mammalian cells by using a CMOS sensor at zero distance between the cell and sensor surface, Tanaka and co-workers [72] performed chemiluminescent imaging of HeLa cells with the CMOS sensor, along with cell staining. This was attained with 7.5 fps frame rate and by using a CMOS sensor. Living and dead cells were successfully distinguished by white- and blue-colored images using trypan blue staining of HeLa cells [72] (Figure 2). The ultimate aim was to develop a miniature cytometer for high throughput cell profiling. Zero-distance imaging by assembling individual cells on micro-lens array of the CMOS sensor was preferred for being advantageous in photon collection efficiency [73]. This brings about the suitability of CMOS sensor for real-time and high-content analysis of single cells.

An interesting study to be mentioned here is the utilization of a dual active pixel sensor (APS)-array scheme to facilitate the determination of the velocity and size of particles flowing in microfluidic channels, with the use of a CMOS optical APS [74]. The prototype dual-APS sensor was informed to be capable of detecting particle velocities up to approximately 500 μm/s and particles with diameter in the range of 5–15 μm [74]. The dual APS CMOS sensor was not demonstrated with cells, but was offered as an integrated optical sensor for cell detection and analysis. Point-of-care diagnostics applications would obviously be possible once the device is demonstrated with cells.

#### 2.2.1. Lens-free Ultra-Wide-Field Cell Monitoring Array Platform Based on Shadow Imaging (LUCAS)

**Figure 3.**
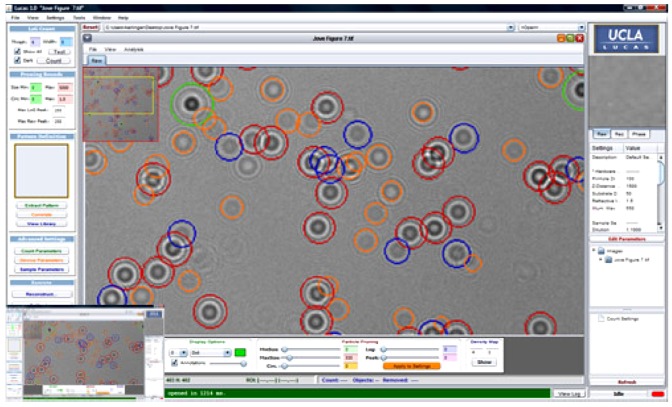
The LUCAS custom interface is shown [76]. Reproduced with the permission of Journal of Visualized Experiments.

**Figure 4.**
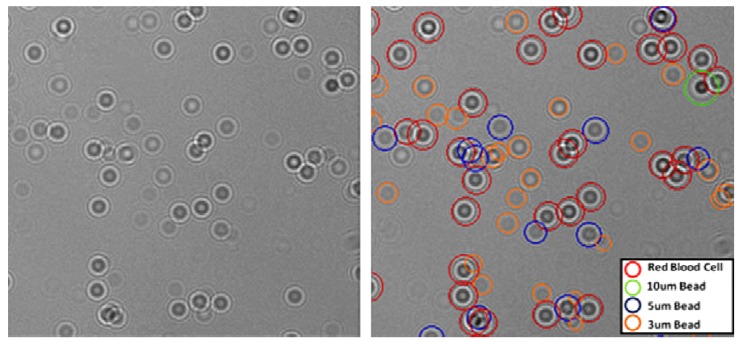
Unprocessed image of a heterogeneous mixture that includes red blood cells and microparticles (10 μm, 5 μm and 3 μm) is shown on the left [76]. Fully-automated LUCAS characterization outcome for the same field of view is demonstrated on the right [76]. Reproduced with the permission of Journal of Visualized Experiments.

A significant progress in the field of CMOS cell imaging for point-of-care diagnostics is named LUCAS, in short. This approach has the potential to bring the point-of-care diagnostics into reality, not only at hospitals but at under-developed districts and at homes, through evolving individual cell phones into blood analysis tools. The imaging method was realized by projecting the diffraction patterns of cells directly onto 2D photosensors such as CMOS sensor [37,75]. The light source is not required to be a laser and therefore even a simple light emitting diode (LED) can be used for illumination [76]. CMOS-based imaging platform records the shadows such as lensless digital holograms [76]. Then, automated digital processing with custom-developed LUCAS decision software (Figure 3) determines the type, count, and relative positions of cells within the heterogeneous cell population in solution (Figure 4). Its advantage over lens-based microscopy is the large field-of-view and its being capable of operation without scanning stages and optical components such as lenses. Yet, the resolution is at the cell-size level, so single virus particles cannot be directly visualized.

##### An Improvement: Holographic-LUCAS

In lens-free holographic imaging, signal to noise ratio and unique features of cells were further developed along with considerable progress in differentiating different cell types [75]. The basis is as follows: incoherent LED light is initially filtered by passing it through a large aperture of about 50–100 μm diameter. After propagating in air a distance of about 3–4 cm, the light interacts with the object of interest [77]. The interference of the light waves, which had passed through the cells, with the unscattered LED light creates the hologram of each object with unit fringe magnification. The hologram is detected without any lens, only by using a CMOS sensor array [77]. The improvements enabled label-free characterization of a heterogeneous cell population on a chip over a field of view of about 10 cm^2^ and a depth of field of more than 4 mm, which is corresponding to a solution volume of more than 4 mL [75]. Due to its ultra-large field of view, holographic-LUCAS can monitor more than 100,000 cells in parallel, for a cell density of about 3,000 cells mL^−1^. With improved performance of the Holographic-LUCAS platform, sensor imaging of phase objects such as *E. coli* samples can be designed [75]. Such samples are hard to be recognized under a 40× objective lens of the microscope [75].

### 2.3. Imaging Sensors with Dual Functionalities for Bioscientific Sensing

**Figure 5.**
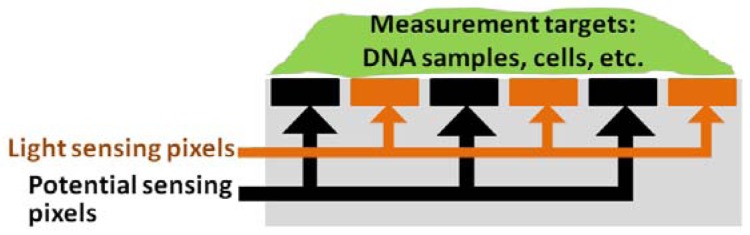
Concept of the optical and potential dual image CMOS sensor [78]. The actual sensor consisted of a 176 × 144 pixel array with 7.5 × 7.5 μm pixel sizes, and 88 columns were spared for optical-sensing and the rest 88 for potential-sensing [78]. Adapted with the permission of Elsevier.

While the imaging concept with CMOS sensors was being improved with studies such as holographic-imaging, multiplicity of functions were attempted to be combined on a single chip. Accordingly, 2D optical and potential dual imaging CMOS sensor was demonstrated for bioscientific applications (Figure 5). The profile of a potential spot having a diameter of less than 50 μm was observed [78]. By choosing an appropriate operating sequence and off-chip configuration, the sensor was capable of operating in either a wide-range potential imaging mode (>5 V) or a high-resolution potential imaging mode (1.6 mV) [78]. The sensor was described and demonstrated to have applications including on-chip optical and potential neural imaging. On-chip biomolecular sensing with fluorescence and potential dual measurement schemes was specifically implemented in sensing deoxyribonucleic acid (DNA) hybridization [78]. The target application of neural imaging was on-chip imaging of the field potentials of a neural system.

**Figure 6.**
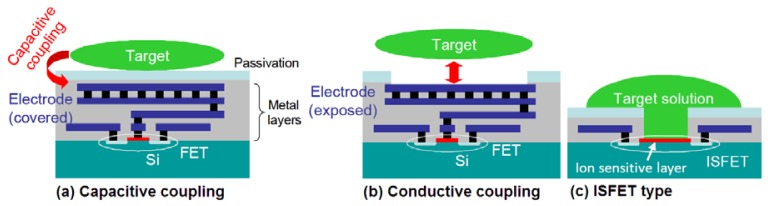
Illustration of the range of concepts and configurations of on-chip “passive” electric sensing that are using the gate of a MOS transistor as the sensing input, namely the capacitive coupling (**a**), conductive coupling (**b**), and ISFET type (**c**) [79]. Reproduced from the Open Access publication Materials.

Recently, Tokuda and co-workers [79] described the APS design that is currently in use in most CMOS image sensors as the only realistic light sensing architecture for multifunctional realization of optical and electric sensing devices. Photodiode structure used in conventional APS circuitry, which is 3-transistor APS, can be replaced with a photogate structure [79]. Alternatively, in some fabrication technologies, a pinned photodiode is available for superior performance (4-transistor APS) [79]. Electric sensing architecture can as well be varied for achievement of multifunctional CMOS image sensors. Capacitive and conductive couplings are possible in the “passive” configuration, wherein only a small amount of capacitive charge transfer occurs between the sensing input and measurement target with no net current flow in between [Figure 6(a,b)]. Ion sensitive field-effect-transistor (ISFET) can be included in this measurement scheme [Figure 6(c)]. These measurement schemes can be realized with circuits as simple as APS.

Voltage application or current injections is not possible by using the passive configuration, but a sensing electrode connected through switching circuitry is required to realize “active” measurement schemes, with voltage applications or current injections from or to the sensing electrode [79]. By means of an active sensing approach, both capacitive coupling (in which the sensing electrode is covered with a dielectric insulation layer) and conductive coupling (with exposed sensing electrodes) can be achieved [79]. Using capacitive coupling through an insulated electrode, voltage can be applied or on-chip potential can be sensed capacitively [79]. With the active electric sensing scheme, on-chip potential profile can be controlled with no net current flow [79]. Multifunctional sensor capable of controlling the positions of living cells cultured on a CMOS image sensor was realized by this means [80,81]. As mentioned, sensors that employed active electric pixels only for voltage application was developed [80–82]. 2D potential distribution was generated with the use of capacitively coupled active electric pixels, to control the positions of cultured cells by dielectrophoresis [80–82]. On the other hand, intelligent multielectrode array devices can be realized by exposing the sensing electrode to detect the extracellular potentials of neural cells [83–85].

**Figure 7.**
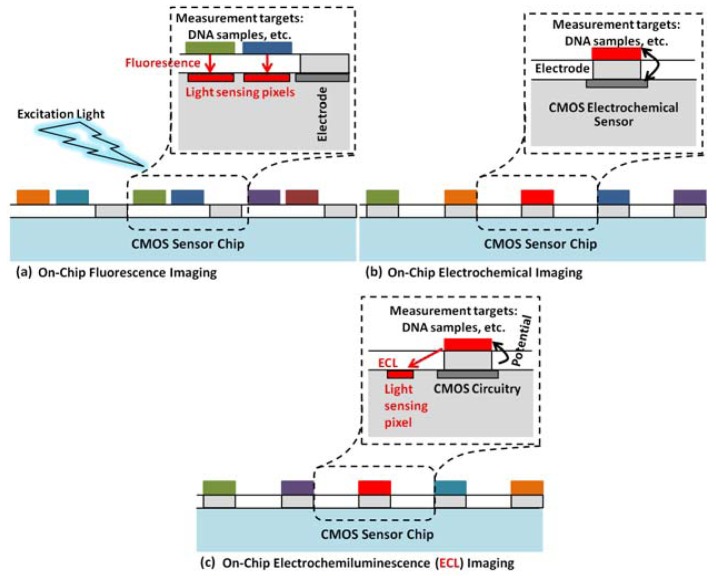
The concept of on-chip biomolecular detection using an optical (**a**) and an electrochemical (**b**) sensor combined in a dual-image CMOS sensor to detect electrochemiluminesence (**c**) [86]. Adapted with the permission of Elsevier.

**Figure 8.**
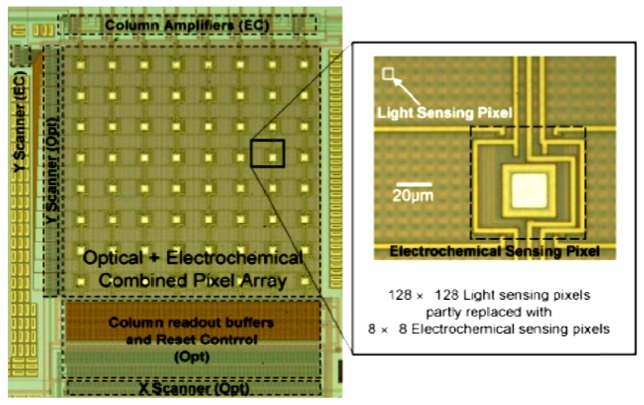
Micrographs of the fabricated optical and electrochemical dual-image CMOS sensor [86]. The combined pixel array was a 128 × 128 CMOS optical image sensor, partly replaced with electrochemical sensing pixels [86]. The sensor had 8 × 8 embedded electrochemical pixel array that were obtained through the substitution of light sensing pixels in the optical image sensor [86]. The light-sensing pixel was a modified three-transistor CMOS active pixel sensor (7.5 × 7.5 μm) [86]. The size of the electrochemical-sensing pixel was 60 × 60 μm and it contained conductively coupled electric sensing electrode (30.5 × 30.5 μm) with a transmission-gate switch for row selection [86]. Mismatch in the operating speed between the optical image sensor and electrochemical image sensor was compensated by designing the optical and the electrochemical pixel arrays to work independently [86]. Reproduced with the permission of Elsevier.

Optical and electrical dual-image sensor was developed for on-chip active electronic measurements with the sensing electrode exposed on the surface in order to establish conductive coupling to the on-chip measurement target [86]. Dual image profile was adapted both to receive image and to perform electrochemical measurements of the sample with a 2D arrayed voltammetry in an on-chip configuration, simultaneously and independently [78]. The sensor was achieved by implementing 8 × 8 pixel array electrochemical sensor into a 128 × 128 active pixel sensor CMOS image sensor, and the current range of the electrochemical measurement function was measured to be approximately 10 nA and 100 μA [86]. In addition to using optical and electrochemical sensing functions complementarily, authors expected to detect electrochemiluminesence in combination, as shown in Figure 7 below. This dual-function chip (Figure 8) was not demonstrated with the cells but the concept well suits for the cell-based studies.

**Figure 9.**
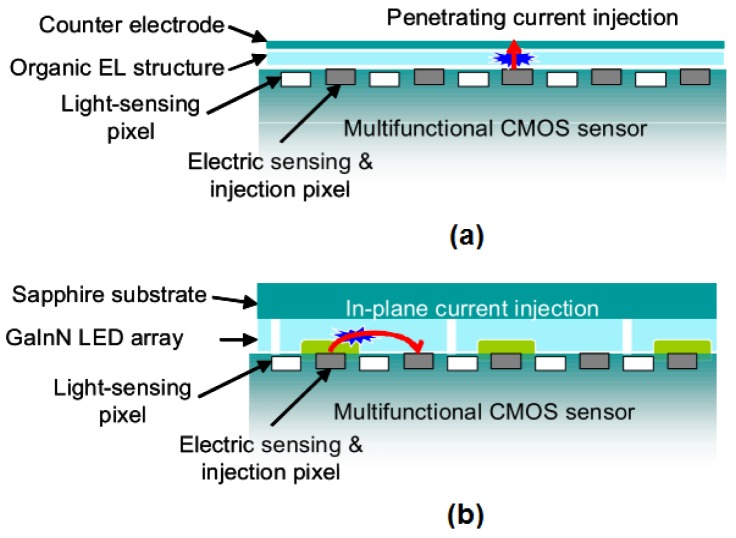
Current paths in the integrated light source with an organic electroluminescence (EL) layer (**a**) and a GaInN LED array (**b**) [79]. Penetrating (single-pixel) current injection that is compatible with the light-emission structure can be achieved, once one of the electric pixel rows is chosen (**a**) [79]. In-plane (inter-pixel) current injection using two current injection lines at the same time can be realized, which is compatible with a light-emission structure that have a horizontal current flow (**b**) [79]. Reproduced from the Open Access publication Materials.

A multifunctional CMOS image sensor for local current injection was described by Tokuda and co-workers [79] with a slight modification of the optical and electric multifunctional CMOS image sensor design, wherein they employed a random-access scheme for electric sensing pixel to be able to select electric pixels for current injection (Figure 9). The concept was developed as a multifunctional biointerface (neural-interface) device with a target field of application in brain/neural science [79].

### 2.4. Imaging Sensors for Testing Drug Effects

Drug discovery pharmacology, neural interface systems, cell-based biosensors and electrophysiology studies are key applications of neuron stimulation and recording [87]. Testing drug effects, which is critical in drug discovery pharmacology, can have potential roles in point-of-care diagnostics, theranostics, and personalized medicine in the future, which could be crucial in cases of drug responsive individuals and emergency conditions in relation.

A CMOS imaging module was developed for examining the drug effects on the beating rate and beat-to-beat variations of embryonic stem cell (ESC)-derived cardiomyocytes that were cultured on a commercially available chamber slide [88]. These cardiomyocytes were measured with a CMOS imaging module, a white LED, and a pinhole. For this work, the authors simply extracted the CMOS imaging module from a conventional webcam and performed lens-free imaging. Real-time image processing was conducted by comparing reference and live frame images, with an image processing algorithm that have been developed by them for analyzing squirming objects like beating cardiomyocytes [88]. The resolution was not high enough to distinguish details of cells, but was sufficient to detect the cardiomyocytes' beating. Cardiotoxic activities of drugs were offered to be tested with such a platform. The indicators of cardiac dysfunction, changes in the beating rate and beat to beat variations were measured in real time under the influence of two different drugs, isoprenaline and doxorubicin [88]. Tachycardia (abnormally increasing heart rate) and bradycardia (abnormally decreasing heart rate) were detected in real-time, respectively, for the tested drugs. Besides, the effectiveness of cardiovascular drugs or the appropriate dosages of the prospective drug candidates can be evaluated with similar platforms. It was informed that 30% of total drugs that were withdrawn from a diversity of major markets between the years 1996 and 2006 were related to cardiac dysfunction [88]. So, these devices look like just the right solutions for preliminary lab-bench testing, and to save both expenditures and lives from the drugs for their unforeseen side-effects.

It can also be mentioned that cell-based studies may be adapted, for example, to test the effect of drugs on the ionic conductivities of cells that are optically imaged in a simultaneous manner during the tests. With the ISFET type configuration, optical and pH multifunctional image sensors can be designed to enable both modalities, based on charge transfer gate structure [79]. For the combined optical and pH dual imaging, gate functioned as a photogate in a light-sensing pixel and as a proton-sensitive ISFET in a pH-sensing pixel [79]. Dual imaging in the same sensing area within the pixel was demonstrated due to the compatibility of light-sensing and pH-sensing pixel [89].

## 3. CMOS-Compatible Silicon Nanowire-Based Sensors for Cell Studies

**Figure 10.**
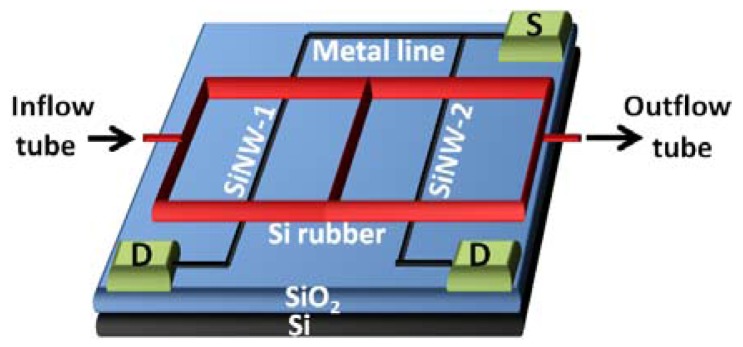
Schematic drawing of silicone nanowire (SiNW) chip dually functionalized with antibodies against tumor necrosis factor-alpha and interleukin-6, respectively at SiNW-1 and SiNW-2 [93]. S denotes source and D denotes drain. Adapted with the permission of Elsevier.

Reliability and sensitivity are among essential criteria of point-of-care diagnostics. Silicon nanowire-based sensors, which can be fabricated by means of CMOS compatible processes, have the potential to improve sensitivities due to their inherent characteristics. Since, silicon nanowires perform as the conducting channel in proximity to the sensing environment. This is analogous to bulk silicon FETs, where the conducting channel is buried under a dielectric insulating layer [90]. One dimensional structure is further benefited for a large surface-to-volume ratio. Sensitivities, which were revealed to be elevated to a level that was sufficient to detect biomarker proteins down to 1 fg/mL in buffer and 30 fg/mL in undiluted human serum [91], are expected to be effective for cell detection as well. Pui and co-workers [92] utilized silicone nanowire-based FETs to detect adipocytokines secreted by adipocytes, to understand the interplay between the adipocytokines and their physiological functions. Aligned arrays of silicone nanowire single cystals were fabricated by means of top-down CMOS-compatible fabrication techniques [92]. Recently, the same authors [93] employed dually-functionalized silicon nanowire chip (Figure 10) for parallel detection of the secretion of pro-inflammatory cytokines, tumor necrosis factor-alpha and interleukin-6, during immune response of macrophages to the stimulation of bacterial endotoxin lipopolysaccharide. The study was indicated to be not only influential for the study of immunity but key to diagnosis or to monitor drug treatment of pathological states as well [93]. While these approaches may be of use for drug research, a diverse approach came earlier from another study of the same group. They performed electrophysiological measurements at the single-cell level or from tissues, which could be an application in high-throughput drug screening for ion channels [90]. Electrocardiogram (ECG) signals from a beating rat heart was measured by detecting cardioelectricity in characteristic changes of their conducting current, and also, spontaneous membrane action potentials from individual rat cardiomyocytes were transduced into changes in conductance [90]. In the former case, the beating heart was placed into the recording chamber on the top of the nanowire chip; while, in the latter case, the cardiomyocytes were isolated from the hearts of neonatal rats and cultured on the nanowire chip for a few days until the experiment [90]. Another type of muscle cell, rat aortic muscle cells (A7R5) were also sensed with the silicon nanowire chip, for their membrane activities and action potentials, by introducing concentrated potassium solution into the recording chamber [90]. This resulted in the observation of current spikes. The authors stated that the local extracellular voltage at the narrow cleft between the adhering membrane patch and the underneath nanowire was transduced into nanowire current signals, indirectly.

Stern and co-workers [94] fabricated silicon nanowires with a CMOS-compatible technology, and performed label-free immunodetection of mouse immunoglobulins G and A, at sub-100 fM levels, as well as real-time monitoring of cellular immune response. Cellular immune response was analyzed through T-lymphocyte activation. A current decrease was detected after about 10 seconds following the addition of species-specific antibody directed against the mouse CD3 complex to a mouse splenocytes suspension, which was bearing T cells [94]. Besides, a control experiment with a species specific antibody to human CD3 resulted in no response. For both diagnostic assessment and planning of therapeutic strategies for many disease states, antigen-specific T-cells recognition is of high importance [95]. Antigen specific T-cell response detection by means of extracellular release of protons upon activation by triggering the T-cell antigen receptor was reported two years later, by employing CMOS technology [95]. The cells were splenocytes that were isolated from a C57BL/6 (B6) mouse and suspended in a low-buffered solution. Stimulation was performed with anti-CD3 antibody. The technique was offered to have the potential to unravel the kinetics of activation-induced T-cell responses further. Besides, validating a functional cellular response makes it desirable for development as a point-of-care testing platform for label-free sensing with direct electronic readout to differentiate specific T-cell populations with a size as small as ∼210 cells within seconds [95]. Still, the concept can be expanded to other cellular systems, as the proton secretion is a common signal transduction mechanism [95].

## 4. Conclusions and Outlook

Developments are driving the CMOS sensor technology as surpassing functionality modules of the conventional devices and their counterparts. The multiple task levels that can be performed with a single CMOS sensor have the potential to follow this boosted scheme. State of the art CMOS image sensor technology is showing appreciable progress in improving the image quality to achieve high resolution, depth-profiling images. Moreover, they are accumulating diverse functionalities by handling pixels of the APS independently. Lens-free image sensors offer flexible, compact and cost-effective alternative for their conventional counterparts, and they do not rely on optical components such as lenses, mirrors, and beam splitters. Yet, the technique is not a rival of conventional microscopy, which is effectively functioning with the lenses and other optical components, but is perfect for the evolution of simple conventional cell-phones into “laboratories-at-hand” for high-throughput cell-biology and point-of-care medical cytometry for global health related problems. Besides, there is still much to gain from available state of the art improvements in the techniques of conventional systems, for exploring the potential. For example, in case of holographic LUCAS imaging, tunable wavelength illumination provides flexibility to the stage where the holographic signatures of the cells can be adjusted; in addition, hybrid digital signatures can be created, which would improve the signal to noise ratio for superior characterization precision and specificity [76].

In order to be employed in point-of-care testing, CMOS-based cell sensors can be developed to perform, for instance, cellular ionophore measurements, particle and cell sizing, colorimetric cellular biochemical measurements, and real-time measurements of both imaging and specific cell detection. Simultaneous visualization and selective detection can be implemented by adsorbing and imaging whole cells; followed by recognizing specific cell types with cell-specific antibody labeling. Obviously, labeling approaches add an extra step to the detection, but can be unavoidable in situations where specific cell detection cannot be performed by the sizes, contrasts, or signatures of the cell images, or when further biochemical confirmation is necessary to confirm the cell identity. Among several labeling approaches, using cell-specific antibody conjugated quantum dots or labeling cells for chemiluminescent detection can be preferable. Electrochemiluminescent quantum dots do not need any illumination scheme, so are immune from the adverse effects of the need to excite the system by radiation. Similarly, chemiluminescence detection is preferable to fluorescence imaging for the reason that the ghost image caused by the excitation light needs to be avoided in case of fluorescence imaging [78]. CMOS sensor has sufficient sensitivity for chemiluminescent imaging of single cells [72]. A relevant concept to chemiluminescence is bioluminescence, where the whole organism is utilized as the bioreporter. The bioluminescence bioreporter integrated circuit (BBIC) utilizes the reaction of a molecule of interest with the genetic regulatory system in a genetically modified bacterial cell, which results in bioluminescence, and the detection of this bioluminescence by CMOS photodetectors [96,97]. The advantage of the technique is that the process is not destructive for the bioreporter organism and nonstop monitoring in real time is enabled [96]. Various methods were described for the bioreporter entrapment at or near the photon-sensing region of the integrated circuit, ranging from entrapment behind a porous membrane to encapsulation in (thin film) polymers or small beads [96]. Based on their earlier studies [98], Bolton and co-workers described a CMOS integrated circuit for chemical sensing, which could detect luminescence from 5,000 fully induced *Pseudomonas fluorescens* 5RL bacterial cells [97]. The chip was realized in a 0.5 μm n-well bulk CMOS process [97]. Later, the total power consumption of the entire circuitry was significantly reduced by Islam and co-workers [99]. It was an integrated CMOS microluminometer, which was realized in 0.35 μm CMOS process. This technology has a potential for providing applications not just in environmental and process monitoring or food safety testing purposes, but also in medical diagnostics [99], which would be overlapping point-of-care diagnostics.

Cell-based biosensors can be used to screen the physiological effects of chemicals of interest [100]. Other than its uses in cell biology and environmental monitoring, this feature aids in toxicology and pharmacology. Of course, cellular response times can be various depending on the mechanism of action of the tested molecule, but the approach would be safer than direct testing of the drug combinations on a patient in a critical state of health. In order to serve for point-of-care applications, cell-based biosensors need to be able to be used effectively outside the laboratory, which necessitates overcoming the issues related to sample preparation, maintenance of the biological environment, and integration of the electronics for data collection and analysis [100]. The last issue can be dealt with the CMOS-based technologies. However, the former issues and keeping the environmental milieu in a cell-friendly manner or at least in a state that will allow proper detection of the analyte in question are yet to be fully resolved, so the major obstacle to practical realization of the CMOS-based devices probably lies in the sample handling and preparation, along with the related issues. Bulky equipment can be required for collection, preparation and sometimes, for maintenance of the samples. This is a potential limiting factor. Developing biosensors and lab-on-a-chip devices for realization of point-of-care diagnostic devices as healthcare solutions at homes, schools, offices, and district regions need to be accepted by health authorities. This requires matching of the functionalities and uses of the devices with conventional tests that are performed at medical centers. In case of biological tests, sample quality need to be assured for the reliability of the outcome of the test results and this cannot be underestimated for point-of-care testing. Thus, the main pitfall of a point-of-care device realization will probably be the development of solutions for custom needs of medical laboratories and health centers, rather than the achievement of technological solutions to complex tests. In 2001, DeBusschere and Kovacs offered a cell-cartridge that was composed of CMOS silicon chip, which incorporated two distinct sample chambers of 10 μL volume each and a digital interface, temperature control system, microelectrode electrophysiology system with 128 total sensor channels that had connections to multiplexers, and analog signal buffering. Action potential signals of cardiomyocyte syncytia from HL-1 cell line were measured with this cell-cartridge after growing the anchorage-dependent cells on the microelectrode sensor. The recorded signals were akin to those obtained in the laboratory, in terms of their shape and amplitude [100]. The use of this cartridge and custom electronics was reported to be resulting in more than 20-folds of less power consumption, weight, and the system volume, with increased number of sensor channels.

As mentioned, biocompatibility is of concern when cell-based studies are the topic of interest. Covering aluminium by additional deposition and patterning at the backend requires post-processing [87]. Electroless gold plating is a valuable and simple solution [101] but there can be defects, resulting in galvanic corrosion of the aluminium [87]. Formation of a porous alumina electrode has been investigated and offered as a low-cost neuronal interface design, with potential use in cell-based research, electrophysiology research, drug discovery pharmacology, and neuronal interface systems [87]. It was shown to bear good corrosion resistance and a morphology that resulted in good cell adhesion [87]. The technique has recently been improved such that, the nano-pores were infiltrated with gold and plated with platinum black, after forming the nano-porous alumina (aluminium oxide) by anodizing the aluminum electrodes [62]. Consequently, the CMOS aluminum electrode was converted into a chemically inert, low impedance electrode consisting of aluminium oxide, gold, and high surface area platinum black [62]. In order to test fast electrical activity with such modified electrodes, primary cultures of rat dorsal ganglion cells were plated onto poly-lysine coated CMOS devices and spontaneous activity was recorded [62]. With the use of caco-2 cell line, electric cell-surface impedance sensing was performed and impedance changes associated with cell growth and division were monitored [62]. Besides biocompatibility, constraints such as use of standard CMOS technologies, packaging of chips that would allow useful lifetimes, and simple and low-cost post-processing are required for realization and commercialization of CMOS cell-based sensors [57]. These issues were recently reviewed elsewhere [57].

Another sampling related issue, especially for point-of-care devices, is the need to keep the samples for appreciably long periods of time for future reference. Sample preservation may be required in order to prevent instances that can result in the accusation of device itself for malfunctioning or the physician for a faulty decision, *etc.* The situation could possibly be worse if the treatment based on the results obtained through the use of device leads to deterioration of the patient's health. Therefore, biological samples of the patient, which would be collected at the same time as testing, may need to be conserved. This obviously seems to be a tough issue, considering the current situation of sensors research and is a challenge for point-of-care devices that are designed to work with bodily specimens. Yet, realization of point-of-care tests with hand-held devices outside laboratories has to overcome such hurdles as well.

Eventually, CMOS-based sensors have an unrevealed potential in bringing novel point-of-care diagnostics technologies into actuality. The concept was tried to be introduced here for cell sensing, in order to draw attention to its potential in fulfilling the needs of novel testing schemes. Yet, there may be lack of demonstrations, which can easily be justified by the under-developed nature of collaborated actions for bringing the concept into reality. So, this hurdle will be overcome; since it is just a matter of time.

## References

[1] Willmott C., Arrowsmith J.E. (2010). Point-of-care testing. Surgery.

[2] Kost G.J., Ehrmeyer S.S., Chernow B., Winkelman J.W., Zaloga G.P., Dellinger R.P., Shirey T. (1999). The Laboratory-Clinical Interface: Point-of-Care Testing. Chest.

[3] Luppa P.B., Müller C., Schlichtiger A., Schlebusch H. (2011). Point-of-care testing (POCT): Current techniques and future perspectives. Trends Anal. Chem..

[4] TriMark Publications (2012). Point of Care Diagnostic Testing World Markets. http://www.trimarkpublications.com/product_images/samples/pocdiagnosticssample.pdf.

[5] Wolters Kluwer Health 2011 Point-of-Care Survey. http://www.wolterskluwerhealth.com/News/Documents/White%20Papers/Wolters%20Kluwer%20Health%20Survey%20Executive%20Summary-Media.pdf.

[6] Jani I.V., Sitoe N.E., Alfai E.R., Chongo P.L., Quevedo J.I., Rocha B.M., Lehe J.D., Peter T.F. (2011). Effect of Point-of-Care CD4 Cell Count Tests on Retention of Patients and Rates of Antiretroviral Therapy Initiation in Primary Health Clinics: An Observational Cohort Study. Lancet.

[7] Lee-Lewandrowski E., Gregory K., Lewandrowski K. (2010). Point of care testing in a large urban academic medical center: Evolving test menu and clinical applications. Clin. Chim. Acta..

[8] Mahieu L., Marien A., de Dooy J., Mahieu M., Mahieu H., van Hoof V. (2012). Implementation of a multi-parameter Point-of-Care-blood test analyzer reduces central laboratory testing and need for blood transfusions in very low birth weight infants. Clin. Chim. Acta..

[9] von Lode P. (2005). Point-of-care immunotesting: Approaching the analytical performance of central laboratory methods. Clin. Biochem..

[10] Satake D., Ebi H., Oku N., Matsuda K., Takao H., Ashiki M., Ishida M. (2002). A sensor for blood cell counter using MEMS technology. Sens. Actuators B Chem..

[11] Tanabe R., Hata S., Shimokohbe A. (2006). MEMS complete blood count sensors designed to reduce noise from electrolysis gas. Microelectron. Eng..

[12] Barrettino D. Design Considerations and Recent Advances in CMOS-Based Microsystems for Point-of-Care Clinical Diagnostics.

[13] Yoo S.J.B. CMOS-compatible Silicon Photonic Integrated Systems in Future Computing and Communication Systems.

[14] St. Louis P. (2000). Status of Point-of-Care Testing: Promise, Realities, and Possibilities. Clin. Biochem..

[15] Chodavarapu V. (2006). Integrated CMOS Sensor Microsystems for Biochemical Monitoring. PhD Thesis.

[16] Boyle D.S., Hawkins K.R., Steele M.S., Singhal M., Cheng X. (2012). Emerging technologies for point-of-care CD4 T-lymphocyte counting. Trends Biotechnol..

[17] Moon S., Keles H.O., Ozcan A., Khademhosseini A., Hæggstrom E., Kuritzkes D., Demirci U. (2009). Integrating microfluidics and lensless imaging for point-of-care testing. Biosens. Bioelectron..

[18] Larsen U.D., Blankenstein G., Branebjerg J. Microchip Coulter Particle Counter.

[19] Mishra N.N., Retterer S., Zieziulewicz T.J., Isaacson M., Szarowski D., Mousseau D.E., Lawrence D.A., Turner J.N. (2005). On-chip micro-biosensor for the detection of human CD4(+) cells based on AC impedance and optical analysis. Biosens. Bioelectron..

[20] Holmes D., Pettigrew D., Reccius C.H., Gwyer J.D., van Berkel C., Holloway J., Davies D.E., Morgan H. (2009). Leukocyte analysis and differentiation using high speed microfluidic single cell impedance cytometry. Lab Chip.

[21] Watkins N., Venkatesan B.M., Toner M., Rodriguez W., Bashir R. (2009). A robust electrical microcytometer with 3-dimensional hydrofocusing. Lab Chip.

[22] Holmes D., Morgan H. (2010). Single cell impedance cytometry for identification and counting of CD4 T-cells in human blood using impedance labels. Anal. Chem..

[23] Jiang X., Spencer M.G. (2010). Electrochemical impedance biosensor with electrode pixels for precise counting of CD4+ cells: A microchip for quantitative diagnosis of HIV infection status of AIDS patients. Biosens. Bioelectron..

[24] Watkins N.N., Sridhar S., Cheng X., Chen G.D., Toner M., Rodriguez W., Bashir R. (2011). A microfabricated electrical differential counter for the selective enumeration of CD4+ T lymphocytes. Lab Chip.

[25] Coulter W.H. High speed automatic blood cell counter and cell size analyzer.

[26] Cheng X., Liu Y.-S., Irimia D., Demirci U., Yang L., Zamir L., Rodríguez W.R., Toner M., Bashir R. (2007). Cell detection and counting through cell lysate impedance spectroscopy in microfluidic devices. Lab Chip.

[27] Yun H., Bang H., Min J., Chung C., Chang J.K., Han D.-C. (2010). Simultaneous counting of two subsets of leukocytes using fluorescent silica nanoparticles in a sheathless microchip flow cytometer. Lab Chip.

[28] Thangawng A.L., Kim J.S., Golden J.P., Anderson G.P., Robertson K.L., Low V., Ligler F.S. (2010). A hard microflow cytometer using groove-generated sheath flow for multiplexed bead and cell assays. Anal. Bioanal. Chem..

[29] Lin Y.-H., Lee G.-B. (2008). Optically induced flow cytometry for continuous microparticle counting and sorting. Biosens. Bioelectron..

[30] Wang M.M., Tu E., Raymond D.E., Yang J.M., Zhang H., Hagen N., Dees B., Mercer E.M., Forster A.H., Kariv I. (2005). Microfluidic sorting of mammalian cells by optical force switching. Nat. Biotechnol..

[31] Mao X., Lin S.C., Dong C., Huang T.J. (2009). Single-layer planar on-chip flow cytometer using microfluidic drifting based three-dimensional (3D) hydrodynamic focusing. Lab Chip.

[32] Church C., Zhu J., Wang G., Tzeng T.-R.J., Xuan X. (2009). Electrokinetic focusing and filtration of cells in a serpentine microchannel. Biomicrofluidics.

[33] Goddard G.R., Sanders C.K., Martin J.C., Kaduchak G., Graves S.W. (2007). Analytical performance of an ultrasonic particle focusing flow cytometer. Anal. Chem..

[34] Zhe J., Jagtiani A., Dutta P., Hu J., Carletta J. (2007). A micromachined high throughput Coulter counter for bioparticle detection and counting. J. Micromech. Microeng..

[35] Wu X., Chon C.H., Wang Y.-N., Kang Y., Li D. (2008). Simultaneous particle counting and detecting on a chip. Lab Chip.

[36] Thorslund S., Larsson R., Nikolajeff F., Bergquist J., Sanchez J. (2007). Bioactivated PDMS microchannel evaluated as sensor for human CD4^+^ cells—The concept of a point-of-care method for HIV monitoring. Sens. Actuat. B Chem..

[37] Ozcan A., Demirci U. (2008). Ultra wide-field lens-free monitoring of cells on-chip. Lab Chip.

[38] Gohring J.T., Fan X. (2010). Label free detection of CD4+ and CD8+ T cells using the optofluidic ring resonator. Sensors.

[39] Li X., Tibbe A.G.J., Droog E., Terstappen L.W.M.M., Greve J. (2007). An immunomagnetic single-platform image cytometer for cell enumeration based on antibody specificity. Clin. Vaccine Immunol..

[40] Wang Z., Chin S.Y., Chin C.D., Sarik J., Harper M., Justman J., Sia S.K. (2010). Microfluidic CD4+ T-cell counting device using chemiluminescence-based detection. Anal. Chem..

[41] Bachelder E.M., Ainslie K.M., Pishko M.V. (2005). Utilizing a quartz crystal microbalance for quantifying CD4(+) T cell counts. Sens. Lett..

[42] Poghossian A., Ingebrandt S., Offenhäusser A., Schöning M.J. (2009). Field-effect devices for detecting cellular signals. Semin. Cell Dev. Biol..

[43] Zheng G., Patolsky F., Cui Y., Wang W.U., Lieber C.M. (2005). Multiplexed electrical detection of cancer markers with nanowire sensor arrays. Nat. Biotechnol..

[44] Patolsky F., Zheng G., Lieber C.M. (2006). Nanowire-based biosensors. Anal. Chem..

[45] Stagni C., Guiducci C., Benini L., Riccò B., Carrara S., Paulus C., Schienle M., Thewes R. (2007). A fully electronic label-free DNA sensor chip. IEEE Sens. J..

[46] Zhang G.-J., Chua J.H., Chee R.-E., Agarwal A., Wong S.M., Buddharaju K.D., Balasubramanian N. (2008). Highly Sensitive Measurements of PNA-DNA Hybridization Using Silicon Nanowire Biosensors. Biosens. Bioelectron..

[47] Levine P.M., Gong P., Levicky R., Shepard K.L. (2009). Real-time, multiplexed electrochemical DNA detection using an active complementary metal-oxide-semiconductor biosensor array with integrated sensor electronics. Biosens. Bioelectron..

[48] Lee K.-H., Choi S.-H., Lee J.-O., Sohn M.-J., Yoon J.-B., Cho G.-H. (2011). An Autonomous CMOS Hysteric Sensor for the Detection of Desorption-Free DNA Hybridisation. Biosens. Bioelectron..

[49] Rothberg J.M., Hinz W., Rearick T.M., Schultz J., Mileski W., Davey M., Leamon J.H., Johnson K., Milgrew M.J., Edwards M. (2011). An integrated semiconductor device enabling non-optical genome sequencing. Nature.

[50] Ng S.Y., Reboud J., Wang K.Y.P., Tang K.C., Zhang L., Wong P., Moe K.T., Shim W., Chen Y. (2010). Label-free impedance detection of low levels of circulating endothelial progenitor cells for point-of-care diagnosis. Biosens. Bioelectron..

[51] Rao L.V., Ekberg B.A., Connor D., Jakubiak F., Vallaro G.M., Snyder M. (2008). Evaluation of a new point of care automated complete blood count (CBC) analyzer in various clinical settings. Clin. Chim. Acta.

[52] Steigert J., Grumann M., Dube M., Streule W., Riegger L., Brenner T., Koltay P., Mittmann K., Zengerle R., Ducrée J. (2006). Direct hemoglobin measurement on a centrifugal microfluidic platform for point-of-care diagnostics. Sens. Actuators A Phys..

[53] Yang Z., Zhou D.M. (2006). Cardiac markers and their point-of-care testing for diagnosis of acute myocardial infarction. Clin. Biochem..

[54] Grosso P., Carrara S., Stagni C., Benini L. (2010). Cancer marker detection in human serum with a point-of-care low-cost system. Sens. Actuators B Chem..

[55] Heer F., Franks W., Blau A., Taschini S., Ziegler C., Hierlemann A., Baltes H. (2004). CMOS microelectrode array for the monitoring of electrogenic cells. Biosens. Bioelectron..

[56] Rossi A.F., Khan D. (2004). Point-of-care testing: Improving pediatric outcomes. Clin. Biochem..

[57] Graham A.H.D., Robbins J., Bowen C.R., Taylor J. (2011). Commercialisation of CMOS Integrated Circuit Technology in Multi-Electrode Arrays for Neuroscience and Cell-Based Biosensors. Sensors.

[58] Fan V., Harman D., Jewett J., Leet B., Speranza D. (2008). Evaluation process for semiconductor fabrication materials that are better for the environment. Intel Technol. J..

[59] Shen L., Ratterman M., Klotzkin D., Papautsky I. (2011). A CMOS optical system for point-of-use luminescent oxygen sensing. Sens. Actuators B Chem..

[60] Prakash S.B., Abshire P. (2008). Tracking cancer cell proliferation on a CMOS capacitance sensor chip. Biosens. Bioelectron..

[61] de Vasconcelos E.A., Peres N.G., Pereira C.O., da Silva V.L., da Silva E.F., Dutra R.F. (2009). Potential of a simplified measurement scheme and device structure for a low cost label-free point-of-care capacitive biosensor. Biosens. Bioelectron..

[62] Graham A.H.D., Surguy S.M., Langlois P., Bowen C.R., Taylor J., Robbins J. (2012). Modification of standard CMOS technology for cell-based biosensors. Biosens. Bioelectron..

[63] Prakash S.B., Abshire P. A CMOS Capacitance Sensor That Monitors Cell Viability.

[64] Prakash S.B., Abshire P. (2007). On-Chip Capacitance Sensing for Cell Monitoring Applications. IEEE Sens. J..

[65] Wigg A.J., Phillips J.W., Wheatland L., Berry M.N. (2003). Assessment of cell concentration and viability of isolated hepatocytes using flow cytometry. Anal. Biochem..

[66] Prakash S.B., Abshire P., Urdaneta M., Smela E. A CMOS Capacitance Sensor for Cell Adhesion Characterization.

[67] Bigas M., Cabruja E., Forest J., Salvi J. (2006). Review of CMOS image sensors. Microelectr. J..

[68] Ji H., Sander D., Haas A., Abshire P.A. CMOS contact imager for locating individual cells.

[69] Ceylan Koydemir H., Kulah H., Ozgen C., Alp A., Hascelik G. (2011). MEMS biosensors for detection of methicillin resistant Staphylococcus aureus. Biosens. Bioelectron..

[70] Kling J. (2006). Moving diagnostics from the bench to the bedside. Nat. Biotechnol..

[71] Ji H., Abshire P.A., Urdaneta M., Smela E. CMOS contact imager for monitoring cultured cells.

[72] Tanaka T., Saeki T., Sunaga Y., Matsunaga T. (2010). High-content analysis of single cells directly assembled on CMOS sensor based on color imaging. Biosens. Bioelectron..

[73] Hatakeyama K., Tanaka T., Sawaguchi M., Iwadate A., Mizutani Y., Sasaki K., Tateishi N., Matsunaga T. (2009). Microfluidic device using chemiluminescence and a DNA-arrayed thin film transistor photosensor for single nucleotide polymorphism genotyping of PCR amplicons from whole blood. Lab Chip.

[74] Hosseini Y., Kaler K.V.I.S. (2010). Integrated CMOS optical sensor for cell detection and analysis. Sens. Actuat. A Phys..

[75] Seo S., Su T.-W., Tseng D.K., Erlinger A., Ozcan A. (2009). Lensfree holographic imaging for on-chip cytometry and diagnostics. Lab Chip.

[76] Mudanyali O., Erlinger A., Seo S., Su T.-W., Tseng D., Ozcan A. (2009). Lensless On-chip Imaging of Cells Provides a New Tool for High-throughput Cell-Biology and Medical Diagnostics. J. Vis. Exp..

[77] Mudanyali O., Tseng D., Oh C., Isikman S.O., Sencan I., Bishara W., Oztoprak C., Seo S., Khademhosseini B., Ozcan A. (2010). Compact, light-weight and cost-effective microscope based on lensless incoherent holography for telemedicine applications. Lab Chip.

[78] Tokuda T., Yamamoto A., Kagawa K., Nunoshita M., Ohta J. (2006). A CMOS Sensor with Optical and Potential Dual Imaging Function for On-Chip Bioscientific Applications. Sens. Actuators A Phys..

[79] Tokuda T., Noda T., Sasagawa K., Ohta J. (2011). Optical and electric multifunctional CMOS image sensors for on-chip biosensing applications. Materials.

[80] Manaresi N., Romani A., Medoro G., Altomare L., Leonardi A., Tartagni M., Guerrieri R. (2003). A CMOS chip for individual cell manipulation and detection. IEEE J. Solid St. Circ..

[81] Romani A., Manaresi N., Marzocchi L., Medoro G., Leonardi A., Altomare L., Tartagni M., Guerrieri R. Capacitive sensor array for localization of bioparticles in CMOS lab-on-a-chip.

[82] Medoro G., Manaresi N., Leonardi A., Altomare L., Tartagni M., Guerrieri R. (2003). A lab-on-a-chip for cell detection and manipulation. IEEE Sens. J..

[83] Heer F., Franks W., McKay I., Taschini S., Hierlemann A., Baltes H. CMOS microelectrode array for extracellular stimulation and recording of electrogenic cells.

[84] Heer F., Hafizovic S., Franks W., Ugniwenko T., Blau A., Ziegler C., Hierlemann A. CMOS microelectrode array for bidirectional interaction with neuronal networks.

[85] Hafizovic S., Heer F., Ugniwenko T., Frey U., Blau A., Ziegler C., Hierlemann A. (2007). A CMOS-based microelectrode array for interaction with neuronal cultures. J. Neurosci. Meth..

[86] Tokuda T., Tanaka K., Matsuo M., Kagawa K., Nunoshita M., Ohta J. (2007). Optical and electrochemical dual-image CMOS sensor for on-chip biomolecular sensing applications. Sens. Actuators A Phys..

[87] Graham A.H.D., Bowen C.R., Robbins J., Taylor J. (2009). Formation of a porous alumina electrode as a low-cost CMOS neuronal electrode. Sens. Actuators B Chem..

[88] Kim S.B., Bae H., Cha J.M., Moon S.J., Dokmeci M.R., Cropek D.M., Khademhosseini A. (2011). A cell-based biosensor for real-time detection of cardiotoxicity using lensfree imaging. Lab Chip.

[89] Nakazawa H., Ishii H., Ishida M., Sawada K. (2010). A fused pH and fluorescence sensor using the same sensing area. Appl. Phys. Express.

[90] Pui T.-S., Agarwal A., Ye F., Balasubramanian N., Chen P. (2009). CMOS-Compatible Nanowire Sensor Arrays for Detection of Cellular Bioelectricity. Small.

[91] Chua J.H., Chee R.-E., Agarwal A., Wong S.M., Zhang G.-J. (2009). Label-Free Electrical Detection of Cardiac Biomarker with Complementary Metal-Oxide Semiconductor-Compatible Silicon Nanowire Sensor Arrays. Anal. Chem..

[92] Pui T.-S., Agarwal A., Ye F., Tou Z.-Q., Huang Y., Chen P. (2009). Ultra-sensitive detection of adipocytokines with CMOS-compatible silicon nanowire arrays. Nanoscale.

[93] Pui T.-S., Agarwal A., Ye F., Huang Y., Chen P. (2011). Nanoelectronic detection of triggered secretion of pro-inflammatory cytokines using CMOS compatible silicon nanowires. Biosens. Bioelectron..

[94] Stern E., Klemic J.F., Routenberg D.A., Wyrembak P.N., Turner-Evans D.B., Hamilton A.D., LaVan D.A., Fahmy T.M., Reed M.A. (2007). Label-free immunodetection with CMOS-compatible semiconducting nanowires. Nature.

[95] Stern E., Steenblock E.R., Reed M.A., Fahmy T.M. (2008). Label-free Electronic Detection of the Antigen-Specific T-Cell Immune Response. Nano Lett..

[96] Simpson M.L., Sayler G.S., Applegate B.M., Ripp S., Nivens D.E., Paulus M.J., Jellison G.E. (1998). Bioluminescent-bioreporter integrated circuits from novel whole-cell biosensors. Trends Biotechnol..

[97] Bolton E.K., Sayler G.S., Nivens D.E., Rochelle J.M., Ripp S., Simpson M.L. (2002). Integrated CMOS photodetectors and signal processing for low-level chemical sensing with the bioluminescence bioreporter integrated circuit. Sens. Actuators B Chem..

[98] Simpson M.L., Sayler G.S., Patterson G., Nivens D.E., Bolton E.K., Rochelle J.M., Arnott J.C., Applegate B.M., Ripp S., Guillorn M.A. (2001). An integrated CMOS microluminometer for low-level luminescence sensing in the bioluminescent bioreporter integrated circuit. Sens. Actuators B Chem..

[99] Islam S.K., Vijayaraghavan R., Zhang M., Ripp S., Caylor S.D., Weathers B., Moser S., Terry S., Blalock B.J., Sayler G.S. (2007). Integrated Circuit Biosensors Using Living Whole-Cell Bioreporters. IEEE T. Circuits Syst..

[100] DeBusschere B.D., Kovacs G.T.A. (2001). Portable cell-based biosensor system using integrated CMOS cell-cartridges. Biosens. Bioelectron..

[101] Chai K.T.C., Hammond P.A., Cumming D.R.S. (2005). Modification of a CMOS microelectrode array for a bioimpedance imaging system. Sens. Actuators B Chem..

